# Accidental Hypothermia in Hospitalized Adults: Risk Factors, Management Strategies, and Clinical Outcomes: A Systematic Review

**DOI:** 10.7759/cureus.103205

**Published:** 2026-02-08

**Authors:** Raja Waqas, Iqra Kalsoom, Muhammad Qaiser Aziz Khan, Shashwat Shetty, Mohammad G. H Suliman, Amandeep Kaur, Shair Bahadar Khan, Bilal Ahmad

**Affiliations:** 1 Regulatory Sciences and Health Safety, Arizona State University, Tempe, USA; 2 Medicine, Liaquat National Hospital, Karachi, PAK; 3 Cardiac Surgery, Liaquat National Hospital and Medical College, Karachi, PAK; 4 Orthopaedics, Hillingdon Hospital, Uxbridge, GBR; 5 General Surgery, University of Kordofan Hospital, El-Obeid, SDN; 6 General Practice, Shri B M Patil Medical College, Bijapur, IND; 7 General Practice, Healthcare Polyclinic, Dammam, Neom, SAU; 8 General Surgery, Dow University of Health Sciences, Civil Hospital Karachi, Karachi, PAK

**Keywords:** accidental hypothermia, hospitalized adults, management strategies, rewarming, risk factors

## Abstract

Accidental hypothermia (AH) in hospitalized adults is an under-recognized condition associated with increased morbidity, mortality, and prolonged hospital stays. Unlike primary AH caused by environmental exposure, inpatient hypothermia is often secondary to acute illness, comorbidities, iatrogenic factors, or medications that impair thermoregulation. We systematically reviewed studies reporting risk factors, management strategies, and clinical outcomes of AH in adults (≥18 years). Five studies, including 1,698 patients, met the inclusion criteria. Common risk factors were advanced age, male sex, alcohol use, reduced functional status, sepsis, endocrine or neurological disorders, and hospital-related exposures such as cool environments or unwarmed intravenous fluids. Management strategies comprised passive external, active external, and active internal rewarming, with ICU monitoring for moderate-to-severe cases. In-hospital mortality ranged from 13% to 26%, with complications including arrhythmias, coagulopathy, infection, and prolonged length of stay; trauma-specific populations had significantly higher mortality (OR ~5.18). These findings highlight the need for early recognition, targeted rewarming, and preventive measures in high-risk inpatients. Future multicenter prospective studies with standardized definitions and predictive models are required to optimize detection, management, and outcomes of AH in hospitalized adults.

## Introduction and background

Accidental hypothermia (AH) is defined as an unintentional reduction in core body temperature below 35°C, resulting from environmental exposure, impaired thermoregulatory mechanisms, or secondary medical conditions, rather than from clinically induced therapeutic hypothermia [1]. AH is broadly categorized as primary AH, caused by direct environmental exposure (e.g., cold weather, immersion), and secondary AH, occurring due to underlying medical illnesses, metabolic/endocrine disorders, or drug-related impairment of thermoregulation. In clinical practice, AH is further classified by severity as mild (35-32°C), moderate (32-28°C), and severe (<28°C) based on core temperature thresholds, which reflect increasing physiological instability. Although historically described in outdoor, trauma, or wilderness contexts, AH also occurs in hospitalized patients, where it is often underrecognized [2]. In the inpatient setting, reduced mobility, altered consciousness, comorbidities, and iatrogenic factors contribute to dysregulated heat balance and vulnerability to hypothermia.

Hospitalized adults, particularly older and medically complex patients, represent a uniquely at-risk population for hypothermia. Age-related physiological decline, endocrine disorders (e.g., hypothyroidism, adrenal insufficiency), sepsis, neurological impairment, sedative medications, and malnutrition reduce thermogenic capacity and compromise homeostasis. Additionally, hospital environments expose patients to perioperative heat loss, cool procedural areas, inadequate thermal insulation, and intravenous infusions of unwarmed fluids, further compounding risk [3].

The physiological consequences of AH are clinically significant. Even modest reductions in temperature can impair myocardial conduction, precipitate arrhythmias, exacerbate coagulopathy, reduce immune responsiveness, and alter drug metabolism [4]. In hospitalized adults, these pathophysiological effects may amplify existing disease severity, increase intensive care utilization, prolong hospital stays, and contribute to morbidity and mortality. Despite these implications, AH remains widely underreported, inconsistently screened for, and lacking standardized prevention and management protocols across hospital systems.

The primary aim of this systematic review is to synthesize current evidence on the risk factors contributing to AH in hospitalized adult patients. The secondary aims are to summarize reported clinical management strategies and to analyze short- and long-term clinical outcomes associated with AH, including complications, length of stay, and mortality.

## Review

Materials and methods

Search Strategy

The literature search was conducted in four electronic databases, such as PubMed, Embase, Scopus, and the Cochrane Library, from 01 August 2025 to 15 December 2025 to identify studies reporting on accidental or hospital-acquired hypothermia in adult patient populations. A combination of Medical Subject Headings (MeSH) and free-text terms was used, including “accidental hypothermia,” “hospital-acquired hypothermia,” “inpatient hypothermia,” “secondary hypothermia,” “rewarming,” “clinical outcomes,” and “mortality.” Boolean operators (AND/OR) were applied to refine and broaden the search strategy, and filters were used to restrict results to human studies involving adults (≥18 years) (Table 1). Reference lists of all eligible articles and relevant reviews were also manually screened to identify additional studies not captured by the electronic search. English-language articles were included in the final review. The full search strategy was designed to ensure comprehensive identification of observational and interventional studies reporting risk factors, management strategies, and clinical outcomes associated with AH in hospitalized adults. Study identification, screening, eligibility assessment, and inclusion followed the Preferred Reporting Items for Systematic Reviews and Meta-Analyses (PRISMA) guidelines, with a PRISMA flow diagram illustrating the selection process [5].

**Table 1:** Database-Specific Search Strategies for Accidental Hypothermia in Hospitalized Adults AH: accidental hypothermia; MeSH: medical subject headings; ED: emergency department; ICU: intensive care unit; PRISMA: Preferred Reporting Items for Systematic Reviews and Meta-Analyses.

Database	Date Range	Search Terms/MeSH	Boolean Operators/Filters	Notes
PubMed	01 Aug 2025-15 Dec 2025	"accidental hypothermia" OR "hospital-acquired hypothermia" OR "inpatient hypothermia" OR "secondary hypothermia" OR "rewarming" OR "clinical outcomes" OR "mortality"	AND/OR used to combine terms; filters: humans, adults ≥18 years	Reference lists manually screened for additional studies
Embase	01 Aug 2025-15 Dec 2025	"accidental hypothermia"/exp OR "hospital-acquired hypothermia" OR "inpatient hypothermia" OR "secondary hypothermia" OR "rewarming" OR "clinical outcomes" OR "mortality"	AND/OR applied; human studies, adults ≥18 years	Duplicates removed before screening
Scopus	01 Aug 2025-15 Dec 2025	TITLE-ABS-KEY("accidental hypothermia" OR "hospital-acquired hypothermia" OR "inpatient hypothermia" OR "secondary hypothermia" OR "rewarming" OR "clinical outcomes" OR "mortality")	AND/OR applied; human studies, adult patients ≥18 years	Manual reference checking of relevant articles
Cochrane Library	01 Aug 2025-15 Dec 2025	"accidental hypothermia" OR "hospital-acquired hypothermia" OR "inpatient hypothermia" OR "secondary hypothermia" OR "rewarming" OR "clinical outcomes" OR "mortality"	Filters: adults ≥18 years, humans	Additional studies identified from references of included systematic reviews

Eligibility Criteria

Eligibility for inclusion in this systematic review was defined using the PICO framework [6]. The population of interest included hospitalized adult patients aged 18 years or older who experienced AH in medical, surgical, or trauma inpatient settings, including both general wards and intensive care units. Studies focusing exclusively on pediatric populations or healthy outdoor populations were excluded. The exposure or intervention considered encompassed any identified risk factors or clinical events leading to AH, such as environmental, iatrogenic, metabolic, endocrine, neurological, or pharmacological causes. Comparators included patients without hypothermia or those receiving different management strategies for hypothermia, allowing evaluation of outcomes such as mortality, length of hospital stay, and complications; studies without a comparator were also included if they reported relevant outcomes. Outcomes of interest included clinical endpoints such as mortality, intensive care unit admission, duration of hospitalization, complications (including arrhythmias, coagulopathy, and sepsis), and effectiveness of management strategies, including passive external, active external, and active internal rewarming. Eligible study designs comprised observational studies (retrospective or prospective cohorts), registry-based studies, and interventional studies reporting on risk factors, management approaches, or clinical outcomes associated with AH in hospitalized adults. Studies excluded were case reports, reviews, editorials, conference abstracts, pediatric studies, therapeutic hypothermia, outdoor/wilderness-only exposures, and animal studies.

Study Selection

The study selection process followed the PRISMA 2020 guidelines to ensure a systematic and reproducible approach. Initially, all records identified through electronic database searches were screened for relevance based on titles and abstracts. Duplicates were removed, and the remaining records underwent full-text assessment to determine eligibility according to pre-defined inclusion and exclusion criteria. Studies were excluded if they involved pediatric populations, therapeutic hypothermia, outdoor-only exposures, or did not report relevant outcomes in hospitalized adults. After this rigorous screening process, the final set of studies included in the review comprised observational cohorts, prospective studies, and registry-based analyses that reported on risk factors, management strategies, and clinical outcomes of AH in hospitalized adult patients. A PRISMA flow diagram illustrates the identification, screening, eligibility assessment, and inclusion process.

Data Extraction

Data were independently extracted using a standardized form, including study author, year, country, study design, population characteristics, type and severity of hypothermia, identified risk factors, management approaches, and clinical outcomes (mortality, ICU admission, complications, and length of stay).

Risk-of-Bias Assessment

The methodological quality of included studies was assessed using the Newcastle-Ottawa Scale (NOS) for cohort studies, evaluating selection, comparability, and outcome domains [7]. Additionally, the systematic review methodology itself was evaluated using the AMSTAR (A Measurement Tool to Assess Systematic Reviews) 2 checklist, which examines the rigor of literature search strategies, study selection, data extraction, risk-of-bias assessment, and data synthesis [8]. Each study and review was classified as low, moderate, or high risk of bias based on the combination of these assessments, providing a structured framework for evaluating the reliability and validity of the evidence.

Data Synthesis

Due to heterogeneity in study design, populations, and outcome measures, a narrative synthesis was performed, summarizing patterns of risk factors, management strategies, and clinical outcomes.

Registration

This systematic review was not registered with PROSPERO. However, the review was conducted and reported following the PRISMA 2020 guidelines, ensuring a systematic, transparent, and reproducible methodology for study selection, data extraction, and synthesis.

Result

Study Selection Process

**Figure 1:**
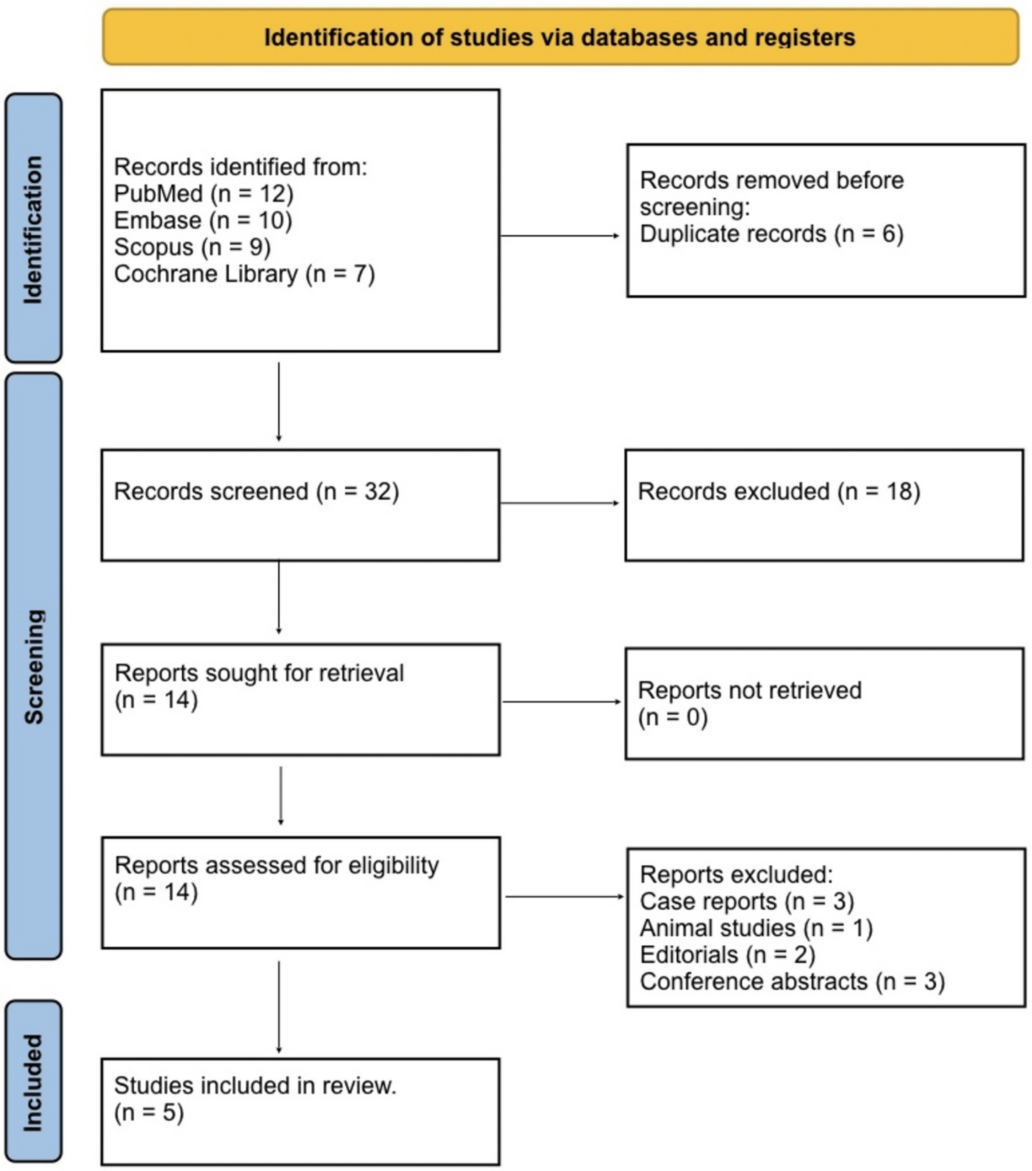
PRISMA 2020 Flow Diagram PRISMA: Preferred Reporting Items for Systematic Reviews and Meta-Analyses.

Figure 1 shows the total of 38 records from electronic databases, including PubMed (n = 12), Embase (n = 10), Scopus (n = 9), and the Cochrane Library (n = 7). After removing six duplicate records, 32 titles and abstracts were screened for relevance. Of these, 18 records were excluded because they did not meet the inclusion criteria, including studies on pediatric populations, therapeutic hypothermia, outdoor-only exposures, or irrelevant outcomes. Full-text versions of 14 studies were assessed for eligibility. Of these, nine studies were excluded due to reasons such as case reports (n = 3), animal studies (n = 1), editorials (n = 2), and conference abstracts (n = 3). Ultimately, five studies met the inclusion criteria and were included in the systematic review, comprising observational cohorts, prospective studies, and registry-based analyses reporting on risk factors, management strategies, and clinical outcomes of AH in hospitalized adults.

Characteristics of the Selected Studies

Table 2 shows five key studies examining AH in hospitalized adults. Takauji et al. (2021) analyzed a multicenter Japanese registry of adults aged ≥18 years (n≈1194) with core temperatures ≤35°C, reporting a 30-day mortality of 24.5%, ICU stay, and hospital length of stay; severe hypothermia was associated with cardiopulmonary arrest, and metabolic and neurologic compromise, with risk factors including age ≥75 years, male sex, low Glasgow Coma Scale, elevated potassium, and ADL (activities of daily living) dependency; common causes were indoor onset, acute illness, trauma, and alcohol, managed with rewarming and supportive care in the ED/ICU [9]. Pirnes and Ala-Kokko (2017) studied 105 adults admitted with AH at Oulu University Hospital, reporting outcomes such as length of stay, complications, and mortality (~13%); pathophysiological findings included lower temperature, acidosis, platelet dysfunction, and altered consciousness, with risk factors like alcohol abuse, lower temperature, and severe acidosis; hypothermia causes included alcohol intoxication and environmental exposure, managed with passive and active rewarming and treatment of complications [10]. Pirnes et al. (2025) evaluated 241 adults with AH, comparing survivors versus non-survivors and reporting 85% 30-day survival; lower admission temperatures were associated with mortality, particularly with indoor exposure; risk factors included alcohol abuse and age ≥65, with causes including indoor versus outdoor exposure, trauma, and submersion, managed with supportive care, prehospital interventions, and rewarming [11]. Okada et al. (2019) focused on 358 adults with moderate-to-severe AH (body temperature ≤32°C), reporting in-hospital mortality of 26.3%; pathophysiological findings included hemodynamic instability and metabolic disturbances, with risk factors including age ≥75 years, hyperkalemia, and hemodynamic compromise; causes were indoor, acute illness, or environmental, managed with ICU rewarming and hemodynamic support [12]. Finally, Rösli et al. (2020) conducted a systematic review of trauma patients across 14 studies, comparing hypothermic patients on admission versus normothermic counterparts; mortality was significantly higher in hypothermic patients (OR ~5.18), with coagulopathy, acidosis, and cardiac depression as major pathophysiological findings; risk factors included injury severity, shock, and transfusion, with trauma and hemorrhage as primary causes, managed with warmed fluids, active rewarming, and extracorporeal membrane oxygenation (ECMO) in severe cases [13].

**Table 2:** Characteristics of the Selected Studies AH: accidental hypothermia; BT: body temperature; ED: emergency department; ICU: intensive care unit; LOS: length of stay; GCS: Glasgow coma scale; ADL: activities of daily living; OR: odds ratio; ECMO: extracorporeal membrane oxygenation.

Authors & Year	Population (P)	Exposure/Condition (I)	Comparator (C)	Outcomes (O)	Pathophysiological Findings	Risk Factors	Accidental Hypothermia Cause	Management/Interventions
Takauji S et al., 2021 [9]	Adults ≥18 years with accidental hypothermia from multicenter Japanese registry (n≈1194)	Core temp ≤35°C (AH)	N/A	30-day mortality (24.5%), ICU stay, hospital stay	Severe hypothermia associated with cardiopulmonary arrest, metabolic and neurologic compromise	Age ≥75 years, male, low GCS, high potassium, ADL dependency	Indoor onset, acute medical illness, trauma, alcohol	Rewarming, supportive care in ED/ICU
Pirnes J et al., 2017 [10]	Adults admitted with AH at Oulu University Hospital (n=105)	Temp ≤35°C	Short stay vs long stay	LOS, complications, mortality (~13%)	Lower temperature, acidosis, platelet dysfunction, altered consciousness	Alcohol abuse, lower temperature, severe acidosis	Alcohol intoxication, environmental exposure	Passive/active rewarming, treat complications
Pirnes J et al., 2025 [11]	Adults admitted with AH (n=241)	Accidental hypothermia	Survivors vs non-survivors	30-day survival (85% survivors)	Lower admission temperatures associated with mortality; indoor exposure worse	Alcohol abuse, age ≥65 years	Indoor vs outdoor exposure, trauma, submersion	Supportive care, prehospital care, rewarming
Okada Y et al., 2019 [12]	Adults with moderate/severe AH (BT ≤32°C, n≈358)	Severe accidental hypothermia	Survivors vs non-survivors	In-hospital mortality (26.3%)	Hemodynamic instability, metabolic disturbances	Age ≥75 years, hyperkalemia, hemodynamic instability	Indoor, acute illness, environmental	Rewarming (ICU), hemodynamic support
Rosli D et al., 2020 [13]	Trauma patients across 14 studies	Hypothermia on admission vs normothermia	Normothermic trauma patients	Mortality (OR ~5.18)	Coagulopathy, acidosis, cardiac depression	Injury severity, shock, transfusion	Trauma with hemorrhage and exposure	Warmed fluids, active rewarming, ECMO

Risk-of-Bias Assessment Table

Table 3 shows that studies were evaluated using validated tools appropriate for each study design. Takauji et al. (2021), a prospective registry cohort, was assessed using the NOS and rated as low-to-moderate risk of bias due to the large dataset and observational design, although residual confounding could not be excluded [9]. Pirnes and Ala-Kokko (2017) conducted a retrospective single-center cohort study, also assessed with NOS, and was rated as moderate risk because of the lack of adjustment for potential confounders [10]. Similarly, Pirnes et al. (2025) performed a multicenter retrospective cohort study, rated moderate risk with NOS, reflecting limited adjustment for confounding variables despite a broader dataset [11]. Okada et al. (2019), a prospective registry study, was rated low-to-moderate risk using NOS, benefiting from good data capture but lacking intervention controls [12]. Finally, Rösli et al. (2020) conducted a systematic review and meta-analysis, which was evaluated using AMSTAR‑2, and was rated moderate risk due to heterogeneity among included studies and trauma-specific confounding factors [13]. Overall, the included studies demonstrated acceptable methodological quality, but several limitations should be considered when interpreting the findings. While prospective registry studies (e.g., Takauji et al. [9], Okada et al. [12]) benefited from large sample sizes and standardized data collection, residual confounding cannot be entirely excluded, particularly for factors such as comorbidities, environmental exposures, or medication use that were not uniformly reported or controlled. Retrospective single- or multicenter studies (e.g., Pirnes et al. [11]) were susceptible to selection bias and limited adjustment for potential confounders, reducing the internal validity of some associations. The systematic review of trauma patients (Rösli et al. [13]) introduced heterogeneity, as it pooled studies with diverse patient populations, settings, and outcome definitions, limiting comparability to general hospitalized adults. Additional sources of bias include variable definitions of AH, differences in rewarming protocols, and inconsistent reporting of severity and clinical outcomes. Taken together, these factors highlight that while the findings are informative and clinically relevant, they should be interpreted with caution, and generalizability may be limited, particularly across different hospital systems, geographic regions, and patient subgroups.

**Table 3:** Risk-of-Bias Assessment Table NOS: Newcastle-Ottawa Scale, a tool used to assess the quality of non-randomized cohort and observational studies by evaluating selection, comparability, and outcomes; AMSTAR‑2: a measurement tool to assess systematic reviews, used to evaluate methodological quality and risk of bias in systematic reviews and meta-analyses; Low-moderate risk: indicates generally reliable study results with minor limitations, such as residual confounding or lack of intervention controls; Moderate risk: indicates potential bias due to factors like single-center design, limited adjustment for confounders, heterogeneity, or trauma-specific confounding.

Study	Design	Risk-of-Bias Tool	Risk-of-Bias Rating	Justification
Takauji S et al., 2021 [9]	Prospective registry cohort	NOS	Low-moderate	Large dataset, observational, residual confounding
Pirnes J et al., 2017 [10]	Retrospective cohort	NOS	Moderate	Single center, no confounder adjustment
Pirnes J et al., 2025 [11]	Retrospective cohort	NOS	Moderate	Multicenter but limited adjustment
Okada Y et al., 2019 [12]	Prospective registry	NOS	Low-moderate	Good data capture, no intervention control
Rösli D et al., 2020 [13]	Systematic review and meta‑analysis	AMSTAR‑2	Moderate	Heterogeneity, trauma-specific confounding

Discussion

AH in hospitalized adults remains an underrecognized, yet clinically significant, condition, with implications for morbidity, mortality, and healthcare resource utilization. Our systematic review highlights that AH occurs not only due to environmental exposure but also secondary to medical illnesses, iatrogenic factors, and pharmacological agents. Primary AH, caused by direct exposure to cold environments, is less common in hospitalized populations, whereas secondary AH, arising from acute illness, endocrine/metabolic disorders, neurological impairment, or drug-induced thermoregulatory dysfunction, predominates in inpatient settings. Across the included studies, both community-acquired indoor hypothermia and hospital-acquired hypothermia were reported, emphasizing the need for vigilance in monitoring core temperature, especially in patients with limited mobility, cognitive impairment, or altered consciousness [9,13].

The pathophysiology of AH in hospitalized adults involves a complex interplay of impaired thermogenesis, altered cardiovascular responses, and metabolic derangements. Even mild reductions in body temperature can disrupt enzymatic activity, reduce myocardial conduction, impair coagulation, and suppress immune function. Severe hypothermia is associated with hemodynamic instability, metabolic acidosis, electrolyte disturbances (e.g., hyperkalemia), and neurological compromise, as observed in registry and cohort studies [9,12]. In trauma populations, hypothermia exacerbates the “lethal triad” of coagulopathy, acidosis, and hypothermia, leading to significantly increased mortality compared with normothermic patients [13]. These findings underscore the importance of early recognition and targeted interventions to prevent physiologic deterioration in both medical and surgical inpatient populations.

Several risk factors for AH were consistently identified, including advanced age (≥65-75 years), male sex, alcohol use, reduced ADL independence, and comorbidities such as sepsis, endocrine disorders, and neurological impairment.

Iatrogenic and hospital-related factors, including exposure to cool procedural environments, unwarmed intravenous fluids, and inadequate thermal insulation, also contribute significantly to inpatient hypothermia [9,12]. Management strategies focus on rewarming, supportive care, and addressing underlying contributors. Passive external rewarming, active external rewarming (e.g., heated blankets), and active internal rewarming (e.g., warmed intravenous fluids, extracorporeal warming in severe cases) were employed across the studies, with ICU monitoring for moderate-to-severe cases [9-13]. Outcomes varied according to severity, patient comorbidities, and timeliness of intervention; in-hospital mortality ranged from 13% to 26%, with trauma-specific studies reporting a higher risk (OR ~5.18) [13]. Complications associated with AH included arrhythmias, coagulopathy, infection, and prolonged hospital or ICU stays.

Limitations of the included studies include heterogeneity in study populations, settings, and hypothermia definitions, which limit generalizability. Several studies were retrospective or single-center, introducing potential confounding and selection bias. The use of registry data, although robust, may lack detailed information on interventions or comorbidity severity. Future research should focus on multicenter prospective studies with standardized hypothermia definitions, systematic risk assessment tools, and evaluation of specific rewarming protocols. Investigating preventive strategies, hospital environmental modifications, and early monitoring algorithms in high-risk inpatient populations could reduce incidence and improve clinical outcomes. Additionally, cost-effectiveness analyses of rewarming interventions and predictive models for AH-related complications may guide clinical practice and policy.

## Conclusions

AH in hospitalized adults is a clinically important, yet often underrecognized, condition, predominantly secondary to acute illness, comorbidities, and iatrogenic factors. Advanced age, alcohol use, impaired functional status, and hospital-related exposures significantly increase risk. The pathophysiological consequences, including hemodynamic instability, coagulopathy, metabolic disturbances, and increased susceptibility to complications, underscore the need for timely recognition and targeted management. Current evidence supports rewarming strategies, passive, active external, and active internal, tailored to severity, with ICU monitoring for moderate-to-severe cases, which can improve survival and reduce complications. However, heterogeneity in study designs, retrospective data, and limited multicenter evidence restrict generalizability. Future research should prioritize standardized definitions, prospective multicenter studies, preventive strategies, and predictive models to optimize detection, management, and outcomes of AH in hospitalized adults.
